# Increased Migration of Monocytes in Essential Hypertension Is Associated with Increased Transient Receptor Potential Channel Canonical Type 3 Channels

**DOI:** 10.1371/journal.pone.0032628

**Published:** 2012-03-16

**Authors:** Zhigang Zhao, Yinxing Ni, Jing Chen, Jian Zhong, Hao Yu, Xingsen Xu, Hongbo He, Zhencheng Yan, Alexandra Scholze, Daoyan Liu, Zhiming Zhu, Martin Tepel

**Affiliations:** 1 Department of Hypertension and Endocrinology, Center for Hypertension and Metabolic Diseases, Daping Hospital, Third Military Medical University, Chongqing Institute of Hypertension, Chongqing, China; 2 Department of Nephrology, Charité, Berlin, Germany; and University of Southern Denmark, Institute for Molecular Medicine, Odense, Denmark; University of Bristol, United Kingdom

## Abstract

Increased transient receptor potential canonical type 3 (TRPC3) channels have been observed in patients with essential hypertension. In the present study we tested the hypothesis that increased monocyte migration is associated with increased TRPC3 expression. Monocyte migration assay was performed in a microchemotaxis chamber using chemoattractants formylated peptide Met-Leu-Phe (fMLP) and tumor necrosis factor-α (TNF-α). Proteins were identified by immunoblotting and quantitative in-cell Western assay. The effects of TRP channel-inhibitor 2–aminoethoxydiphenylborane (2-APB) and small interfering RNA knockdown of TRPC3 were investigated. We observed an increased fMLP-induced migration of monocytes from hypertensive patients compared with normotensive control subjects (246±14% vs 151±10%). The TNF-α-induced migration of monocytes in patients with essential hypertension was also significantly increased compared to normotensive control subjects (221±20% vs 138±18%). In the presence of 2-APB or after siRNA knockdown of TRPC3 the fMLP-induced monocyte migration was significantly blocked. The fMLP-induced changes of cytosolic calcium were significantly increased in monocytes from hypertensive patients compared to normotensive control subjects. The fMLP-induced monocyte migration was significantly reduced in the presence of inhibitors of tyrosine kinase and phosphoinositide 3-kinase. We conclude that increased monocyte migration in patients with essential hypertension is associated with increased TRPC3 channels.

## Introduction

An increased transient receptor potential canonical type 3 (TRPC3) protein expression has been observed both in patients with essential hypertension and in animal models of hypertension [1]–[5]. In patients with hypertension an increased TRPC3 expression has been reported in several tissues including vascular smooth muscle cells and peripheral blood monocytes [1], [2], [5]. It is well established that monocytes play a crucial role in atherogenesis by recruitment to the vessel wall [6]. Monocyte activation, adhesion to the endothelium, and transmigration into the subendothelial space are key events in early pathogenesis of atherosclerosis [7]. Earlier studies from Doerffel et al. indicated that monocyte activation is increased in hypertension [8]. Monocytes from patients with essential hypertension show elevated secretion patterns of pro-inflammatory cytokines, an increased expression of adhesion molecules, and an increased adhesion to vascular endothelial cells [9]. Increased activation of monocytes in hypertension may be due to increased change of cytosolic calcium. TRPC3 channels are non-selective cation channels mediating transplasmamembrane calcium influx [10]. TRPC3 channels are likely candidates to produce increased activation of monocytes. An increased calcium influx through TRPC3 channels may cause increased migration of monocytes. However, the role of TRPC3 for regulating the migration of monocytes has not been investigated to date. In the present study we tested the hypothesis that increased TRPC3 channel expression causes increased migration of monocytes from patients with essential hypertension.

## Results

### Increased migration of monocytes from patients with essential hypertension

**Figure 1:**
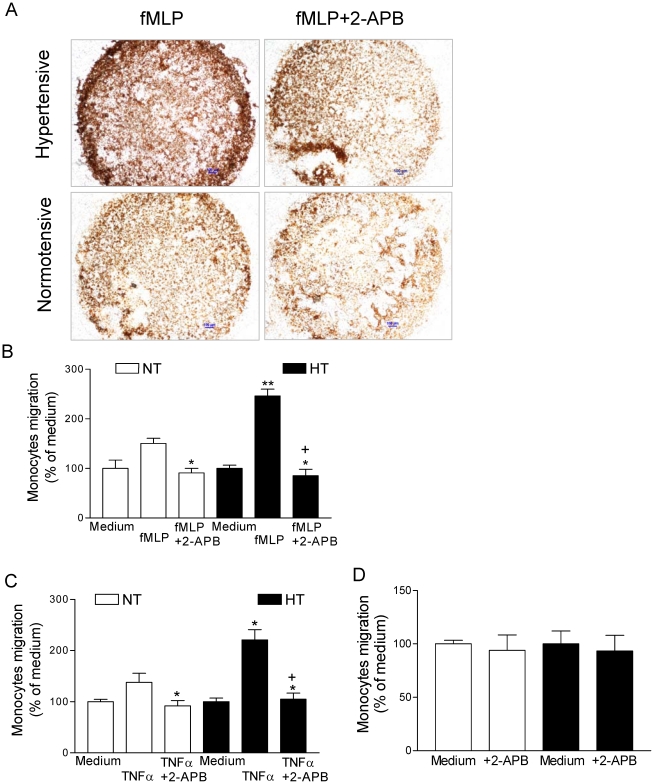
Increased fMLP-induced migration of monocytes from patients with essential hypertension. **A**, Representative microscopy images of the migration of monocytes from hypertensive patients (upper panels) compared with normotensive control subjects (lower panels) in the absence (left panels) or in the presence (right panels) of 2-APB (100 µmol/L). Expreriments were performed in triplicate. Migration was induced by chemoattractant fMLP (100 nm/L). Magnification 4×, bar denotes 100 µm. **B**, **C**, Summary data showing fMLP-induced or TNF-α-induced migration of monocytes from normotensive control subjects (NT; open bars) and hypertensive patients (HT, filled bars) in the absence or presence of 2-APB (100 µmol/L). After the incubation time the polycarbonate filter membranes were dehydrated, stained using fura2-AM and fluorescence was detected at 510 nm emission with 360 nm excitation. Migration rates were normalized to the mean migration rate of monocytes maintained in the medium condition (control). We observed an increased fMLP-induced or TNF-α-induced migration of monocytes from patients with essential hypertension. *p<0.05, **p<0.01 compared to chemoattractant alone in normotensive control subjects, +p>0.05 for the comparison in the presence of 2-APB NT vs. HT. Each n = 10 or 11. **D**; Spontaneous migrations of monocytes from normotensive control subjects (NT; open bars) and hypertensive patients (HT, filled bars) were tested using medium or in the presence of 2-APB (100 µmol/L). The data was quantified by counting the number of cells that had completely migrated through the membrane in six random high-power fields (HPF, 40×) per well. P>0.05 compared to normotensive control subjects. Data are percent of medium as mean ± SEM of three independent experiments.

First we evaluated the migration of monocytes using the chemoattractants fMLP and TNF-α. **Figure 1A** shows representative images of the fMLP-induced migration of monocytes from normotensive control subjects and patients with essential hypertension. We observed an increased fMLP-induced migration of monocytes in patients with essential hypertension compared to normotensive control subjects (246±14% vs 151±10%, each n = 11, P<0.01, **Figure 1B**). To indicate that TRPC channels were associated with the migration of monocytes we inhibited TRPC channels using 2-APB [11], [12], [13]. In normotensive control subjects 2-APB significantly reduced the fMLP-induced migration to 91±10%, whereas in patients with essential hypertension 2-APB significantly reduced the fMLP-induced migration to 86±13% (each n = 11, P<0.05 compared to their control). The fMLP-induced migration of monocytes was significantly reduced in the presence of 2-APB by 65% in patients with essential hypertension, and 40% in normotensive control subjects. The effect of 2-APB was more pronounced in patients with essential hypertension. In the presence of 2-APB the fMLP-induced migration of monocytes was not significantly different in patients with essential hypertension compared with normotensive control subjects (P>0.05). Furthermore, the TNF-α-induced migration of monocytes in patients with essential hypertension was also significantly increased compared to normotensive control subjects (221±20% vs 138±18%, each n = 10, P<0.05). In the presence of 2-APB the TNF-α-induced migration of monocytes was significantly reduced to 92±10% in normotensive control subjects, and in patients with essential hypertension was significantly reduced to 105±12%, each n = 10, p<0.05 compared to their control conditions, **Figures 1C**. We also evaluated that effect of 2-APB on spontaneous migration of monocytes. Our data showed that 2-APB did not affect monocytes spontaneous migration (P>0.05, **Figures 1D**). Therefore these data may point to a functional role of TRPC channels for an elevated agonist-induced migration of monocytes from patients with essential hypertension.

### Increased fMLP-induced changes of intracellular calcium in monocytes from patients with essential hypertension

**Figure 2:**
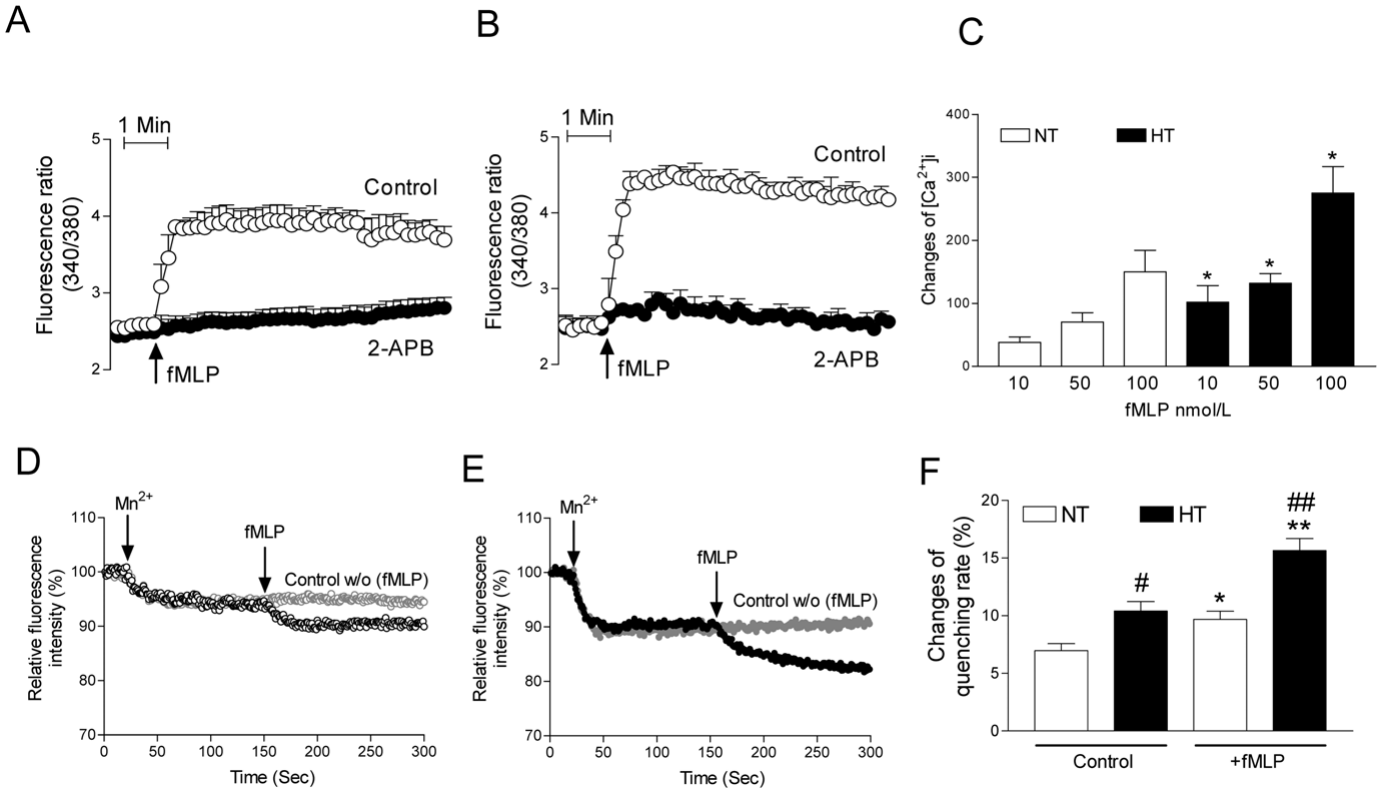
Increased fMLP-induced changes of cytosolic calcium in monocytes from patients with essential hypertension. **A**, **B**; Representative fluorescence tracings in monocytes from normotensive control subjects (**A**) and hypertensive patients (**B**) after administration of fMLP (100 nm/L) in the absence and presence of TRP channel-inhibitor 2-APB. **C**; Summary data of changes of intracellular calcium concentration in monocytes from normotensive control subjects (NT; open bars) and patients with essential hypertension (HT; filled bars) by several doses of fMLP (10 nmol/L, 50 nmol/L, and 100 nm/L). Each n = 10; *p<0.05 for the comparison HT vs. NT. **D**, **E**; fMLP-induced cation influx into monocytes indicated by quenching of fura-2 by manganese (Mn^2+^, 1 µmol/L). Representative raw data, showing mamganese quenching with a stimulus (fMLP, 100 nmol/L) or without stimulus (control, w/o fMLP) in monocytes from normotensive control subjects (**D**; open black or grey circle; NT) and patients with essential hypertension (**E**; filled black or grey circle; HT). **F**; Bar graph shows the summary data of manganese quenching rate obtained from normotensive control subjects (NT, n = 6) and patients with essential hypertension (HT, n = 8) under the control conditions or after administration of fMLP (100 nmol/L). *p<0.05 or **p<0.01 for the comparison with their controls; and #p<0.05 or ## p<0.01 for the comparison HT (filled bars) vs. NT (open bars).

The fMLP-induced changes of intracellular calcium were almost completely abolished by 2-APB in normotensive control subjects (**Figure 2A**) and patients with essential hypertension (**Figure 2B**). In hypertensive patients the changes of the F340 nm/F380 nm fluorescence ratio, which were obtained 5 minutes after administration of fMLP, were reduced from 4.26±0.21 to 2.62±0.19 in the presence of 2-APB. Furthermore, in normotensive control subjects, these changes were reduced from 3.73±0.15 to 2.83±0.12 in the presence of 2-APB. We established that the fMLP-induced changes of intracellular calcium concentration in human monocytes were dose-dependent (**Figure 2C**). The fMLP-induced changes of intracellular calcium concentration were significantly increased in monocytes from patients with essential hypertension compared to normotensive control subjects (fMLP 100 nmol/L; 275±42 vs. 150±34; fMLP 50 nmol/L; 132±15 vs. 70±15; fMLP 10 nmol/L; 102±26 vs. 38±9; each n = 10, p<0.05). The fMLP-induced (100 nmol/L) Ca^2+^ response was elevated by 83% in patients with essential hypertension compared to normotensive control subjects. Cation influx into monocytes was also measured by quenching of fura-2 by Mn^2+^ by standardized techniques. Stimulation was performed with fMLP. For each curve obtained in the presence of a stimulus, the difference to a curve obtained in the absence of a stimulus was calculated, thus yielding the corrected quenching curve in monocytes from normotensive control subjects and patients with essential hypertension (**Figure 2D, 2E**). Under resting conditions the Mn^2+^ influx was significantly increased in patients with essential hypertension compared to normotensive control subjects (10.4±0.8% vs.7.0±0.6%; NT n = 6; HT n = 8, p<0.05). Furthermore, after stimulation with fMLP the Mn^2+^ influx was significantly increased in patients with essential hypertension compared to normotensive control subjects (15.6±1.0% vs. 9.7±0.7%; NT n = 6; HT n = 8, p<0.01; **Figure 2F**)

### TRPC3 knockdown reduced the agonist-induced migration of monocytes from patients with essential hypertension

**Figure 3:**
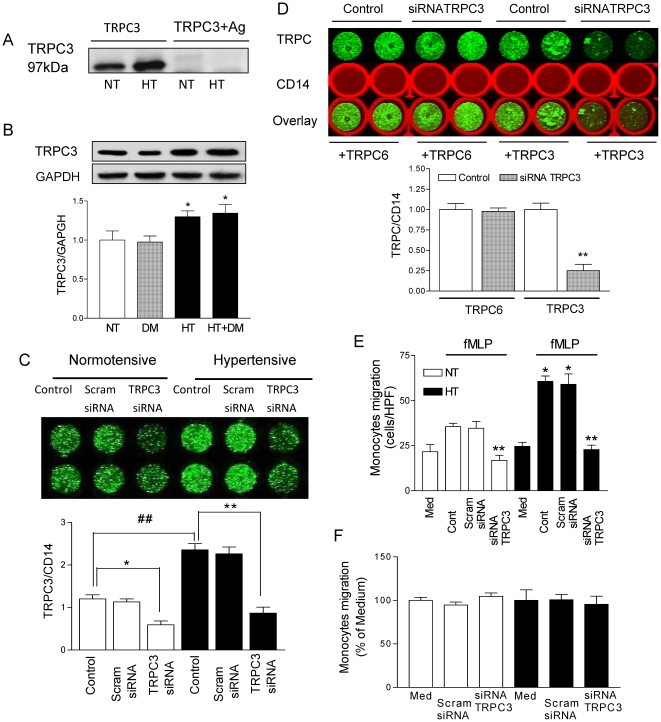
Specific siRNA against TRPC3 blocks the migration in monocytes, but did not affect spontaneous migration of monocytes. **A**; Immunoblot showing specificity of antibodies against TRPC3 in monocytes from normotensive control subjects (NT) and patients with essential hypertension (HT) in the absence or presence of TRPC3 antigens (TRPC3+Ag). The predicted molecular weight of TRPC3 is 97 kDa. **B**; Immunoblot showing specificity of antibodies against TRPC3 in monocytes from normotensive control subjects (NT, n = 8), patients with type 2 diabetes mellitus (DM, n = 9), patients with essential hypertension (HT, n = 8) or hypertensive patients with type 2 diabetes mellitus (HT+DM, n = 10). Summary data of the TRPC3 expression (normalized to GAPDH). *p<0.05, compared to NT. Data are mean ± SEM. **C**; Representative in-cell western assay and summary data of the TRPC3 expression (normalized to CD14 expression used as an internal reference) in monocytes from normotensive control subjects (Normotensive, and opened bars, n = 3) and patients with essential hypertension (Hypertensive, filled bars, n = 3) under control conditions and after transfection with scrambled siRNA or specific siRNA against TRPC3 for 48 h. In-cell western assay was performed using specific antibodies and fluorescence-labeled secondary antibodies. TRPC3 (visible in green) normalized to CD14 (used as an internal reference). Measurements were performed in duplicate for each sample. *p<0.05 or **p<0.01 for the comparison with their controls; and ## p<0.01 for the comparison Hypertensive (filled bars) vs. Normotensive (open bars). **D**; Representative in-cell western assay and summary data of the TRPC3 and TRPC6 expression in monocytes from normotensive control subjects under control conditions and after transfection with specific siRNA against TRPC3 for 48 h. In-cell western assay was performed using specific antibodies and fluorescence-labeled secondary antibodies. TRPC3 and TRPC6 expression (visible in green) normalized to CD14 (visible in red used as an internal reference). Measurements were performed in duplicate for each sample. **p<0.01 compared to control conditions. Data are mean ± SEM of three independent experiments. **E**; Summary data of the fMLP-induced monocyte migration from hypertensive patients (HT, filled bars) and normotensive control subjects (NT, opened bars) quantified by counting the number of cells that had completely migrated through the membrane in six random high-power fields (HPF, 40×) per well. Monocytes chemotaxis was expressed as the mean number of migrated cells per high-power fields from duplicate wells. Experiments were performed under control conditions, after transfection with scrambled siRNA or specific siRNA against TRPC3. *p<0.05; **p<0.01 compared to normotensive control subjects under control conditions. Data are mean ± SEM of eight independent experiments. **F**; Spontaneous migrations of monocytes from normotensive control subjects (NT; open bars) and hypertensive patients (HT, filled bars) were tested using medium or after transfection with scrambled siRNA or specific siRNA against TRPC3. The data was quantified by counting the number of cells that had completely migrated through the membrane in six random high-power fields (HPF, 40×) per well. P>0.05 compared to NT. Data are percent of medium as mean ± SEM of three independent experiments.

The immunoblotting of TRPC3 in monocytes lysates to test the specificity of the antibody for TRPC3 by antigen competition experiments is shown in **Figure 3A**. Using standard western blotting we confirmed increased TRPC3 protein expression in monocytes from both essential hypertensive patients and hypertensive patients with type 2 diabetes mellitus or metabolic syndrome compared to normotensive control subjects (1.30±0.07 for hypertensive patients, N = 8; 1.35±0.11 for hypertensive patients with type 2 diabetes mellitus or metabolic syndrome, N = 10; vs. 1.00±0.11 for normotensive control subjects, N = 8; p<0.05). On the other hand TRPC3 protein expression was not significantly increased in patients with type 2 diabetes mellitus when compared to normotensive control subjects (0.97±0.08 for patients with type 2 diabetes mellitus, N = 9; vs 1.00±0.11 for normotensive control subjects, N = 8 p>0.05), as shown in **Figure 3B**. Additional measurements were preformed in the absence of cell permeabilization to investigate TRP channel protein expression on the surface of the cells. We observed an increased expression of TRPC3 channel proteins on the surface of monocytes by 40% (1.40±0.13 for patients with hypertension, N = 6; vs. 1.00±0.02 for normotensive control subjects, N = 6; p<0.01). Total TRPC3 channel protein expression was also increased in patients with hypertension. These results confirm previous findings from our group showing increased total TRPC3 channel expression on monocytes from patients with essential hypertension (3.21±0.59 for patients with hypertension, N = 20; vs. 1.36±0.07 for normotensive control subjects, N = 20; p<0.05; J Hypertens. 2006). These findings support the idea that increased TRPC channel protein expression in patients with hypertension increases transplasmamembrane calcium influx. Based on our previous study [14], we performed quantitative real-time PCR indicating that siRNA against TRPC3 significantly reduced TRPC3 transcripts to 11±2% of control (p<0.05). To verify that TRPC3 channels are involved in monocyte migration, TRPC3 knockdown was performed by gene silencing with RNA interference using specific siRNA or scrambled siRNA. We found an increased TRPC3 protein expression in monocytes from patients with essential hypertension compared to normotensive control subjects (2.34±0.08 vs. 1.20±0.10; p<0.01). After siRNA knockdown of TRPC3, the TRPC3 expression was not significantly different in monocytes from patients with essential hypertension and normotensive control subjects (0.87±0.14 vs. 0.59±0.10; each n = 8, p>0.05), but there were no effects on TRPC3 expression after scrambled siRNA in monocytes from patients with essential hypertension or normotensive control subjects (**Figure 3C**). After siRNA knockdown of TRPC3 by transfection for 48 hours, the TRPC3 protein expression was significantly reduced from 1.00±0.03 to 0.25±0.08 (p<0.01). There was no effect on TRPC6 protein expression after transfection with siRNA against TRPC3 in monocytes (from 1.00±0.08 to 0.98±0.04; p>0.05, **Figure 3D**). Furthermore, after siRNA knockdown of TRPC3, the fMLP-induced migration was similar in monocytes from patients with essential hypertension and normotensive control subjects (23±3 vs. 17±4; each n = 8, p>0.05; **Figure 3E**) and there were no effects on monocytes migration after transfection with scrambled siRNA in monocytes from patients with essential hypertension or normotensive control subjects. We also evaluated whether TRPC3 channels were involved in spontaneous migration in monocytes. We treated monocytes from normotensive control subjects and patients with essential hypertension with small interfering RNA for knockdown of TRPC3 or scrambled siRNA for control. We observed that specific siRNA against TRPC3 or scrambled siRNA did not significantly affect spontaneous migration of monocytes (**Figure 3F**). This may indicate that TRPC3 does not affect so-called spontaneous migration, but only agonist-induced migration. Our study indicated that the agonist-induced migration is augmented by increased TRP channels in the patients with essential hypertension, whereas spontaneous migration may not be affected.

**Figure 4:**
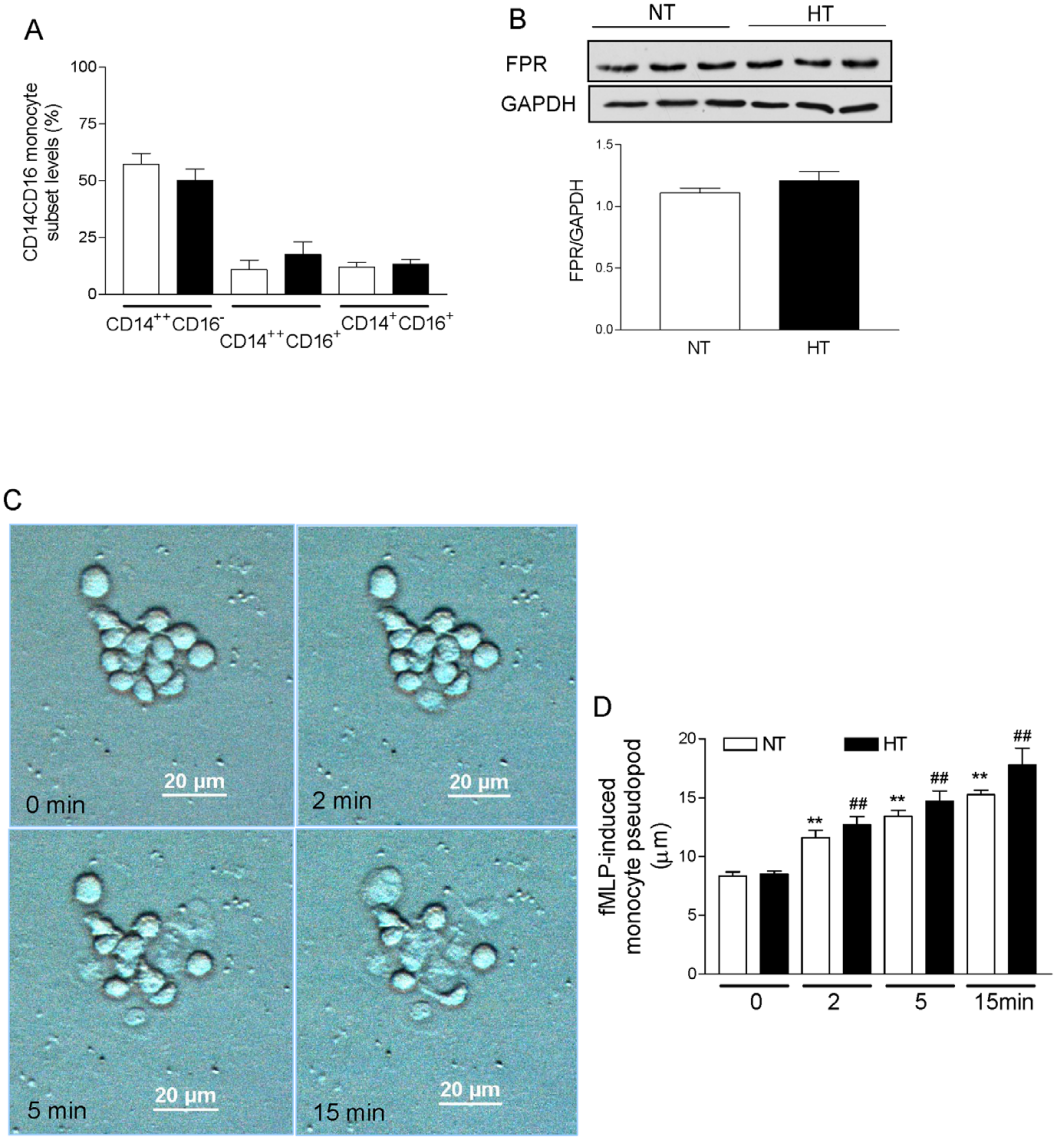
Monocyte subtypes and fMLP receptors in normotensive and hypertensive patients. **A**, Peripheral blood monocytes subpopulations were analyzed by flow-cytometry. After labeling with anti-CD14 phycoerythrin (PE) conjugated and anti-CD16 FITC conjugated, monocytes from patients with essential hypertension (HT, filled bars) and normotensive control subjects (NT; open bars) were readily separated into three distinct subsets according to CD14 and CD16 positivity. Data are mean ± SEM, each n = 11, P>0.05 NT vs. HT. **B**, Expression of fMLP receptors using immunoblotting with specific antibodies. The data showed that fMLP receptors were not significantly different between in monocytes from patients with essential hypertension (HT, filled bars) and normotensive control subjects (NT; open bars). Data are mean ± SEM, n = 6, P>0.05 NT vs. HT. **C**, **D**, Representative micrographs of fMLP-induced polarization response of monocytes (**C**). Summary data of fMLP induced polarization response of monocytes from healthy control subjects (NT; open bars) and from patients with hypertension (HT, filled bars). Data are mean ± SEM, **p<0.01 compared to NT fMLP-stimulation (0 min); ##p<0.01 compared to HT fMLP-stimulation (0 min); P>0.05 NT vs. HT; each n = 12 (**D**).

We evaluated CD14CD16 monocyte subset levels in normotensive control subjects and patients with essential hypertension (Figure 1D). We found that the percentages of CD14^++^CD16^−^ and CD14^+^CD16^+^ subset levels were not significantly differed between normotensive control subjects and patients with essential hypertension (57±6% vs 50±5% for CD14^++^CD16^−^; and 12±2% vs 13±2% for CD14^+^CD16^+^; each n = 11, P>0.05; **Figure 4A**). In addition, the chemotaxis using an incubation time of 4 h was also used to test monocytes' migration. Also for a short incubation time of 4 hours we observed an increased fMLP-induced migration of monocytes from patients with essential hypertension compared to normotensive control subjects (159±12% vs 100±5%; each n = 8, P<0.01). We were interested whether differences of fMLP-induced migration in patients with essential hypertension and healthy subjects might be related to differences in the expression of the receptor for fMLP. Our data showed that fMLP receptors were not significantly different between patients with essential hypertension and normotensive control subjects (P>0.05, **Figure 4B**). These findings indicate that fMLP receptors may not be responsible for the observed differences of fMLP-induced monocytes migration between patients with essential hypertension and normotensive control subjects. On the other hand, microscopy showed that fMLP did not cause significant differences of the polarization response of monocytes from healthy control subjects and patients with hypertension (P>0.05, **Figure 4C, 4D**).

### The role of tyrosine kinase, phosphoinositide 3-kinase and ERK for the migration of monocytes

**Figure 5:**
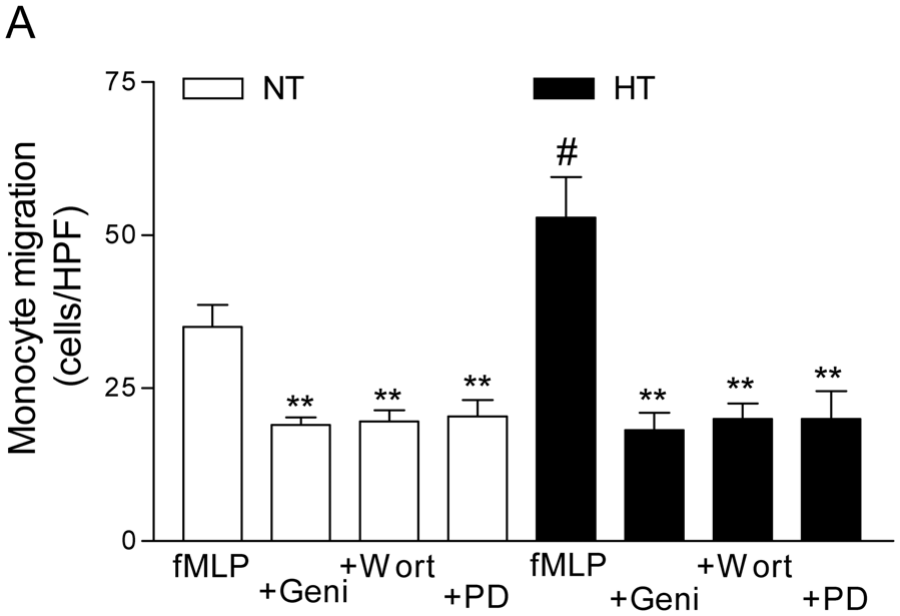
Increased monocyte migration associated with tyrosine kinase and phosphoinositide 3-kinase (PI3K) or ERK in essential hypertension. **A**; Summary data of fMLP-induced monocytes migration was quantified by counting the number of cells that had completely migrated through the membrane in six random high-power fields (HPF, 40×) per well. Monocytes chemotaxis was expressed as the mean number of migrated cells per 40× fields from duplicate wells. Experiments were performed under control conditions (fMLP, n = 6), in the presence of genistein (Geni, n = 6) or wortmannin (Wort, n = 6) and PD98059 (PD, n = 3). Data are mean ± SEM of three to six independent experiments. **p<0.01 compared to their chemoattractant (fMLP) alone; # p<0.05 for comparison HT (filled bars) vs. NT (open bars).

To evaluate the underlying pathways of the migration of monocytes we investigated the roles of tyrosine kinase, phosphoinositide 3-kinase (PI3K) and ERK. As shown in **Figure 5A**, compared to normotensive control subjects, the fMLP-induced migration of monocytes from patients with essential hypertension was significantly increased (mean counts of migrated monocytes under magnification 40×; normotensive control subjects 35±4, patients with essential hypertension 53±7, respectively; p<0.05). On the other hand, the fMLP-induced migration of monocytes was significantly reduced in the presence of the tyrosine kinase blocker, genistein; or PI3K inhibitor, wortmannin; and an inhibitor of ERK, PD98059 (mean counts of migrated monocytes under magnification 40×; genistein 18±3, wortmannin 20±3, and PD98059 20±5 in monocytes from patients with essential hypertension; or genistein 19±1, wortmannin 20±2, and PD98059 20±3 in monocytes from normotensive control subjects; p<0.01 compared to their control conditions). In the presence of the genistein or wortmannin and PD98059, the fMLP-induced migration of monocytes was not significantly different between patients with essential hypertension and normotensive control subjects (p>0.05). Genistein reduced the fMLP-induced migration of monocytes by 46%, and wortmannin reduced it by 43% in normotensive control subjects. Moreover, genistein reduced the fMLP-induced migration of monocytes by 67%, and wortmannin reduced it by 62% in patients with essential hypertension (p<0.01 compared to normotensive control subjects). Furthermore, after siRNA transfection against TRPC3 the effects of genistein or wortmannin could not be observed any longer (22±5; or 21±7 p>0.05 compared with control conditions).

### The role of Akt and ERK-dependent pathways in essential hypertension

**Figure 6:**
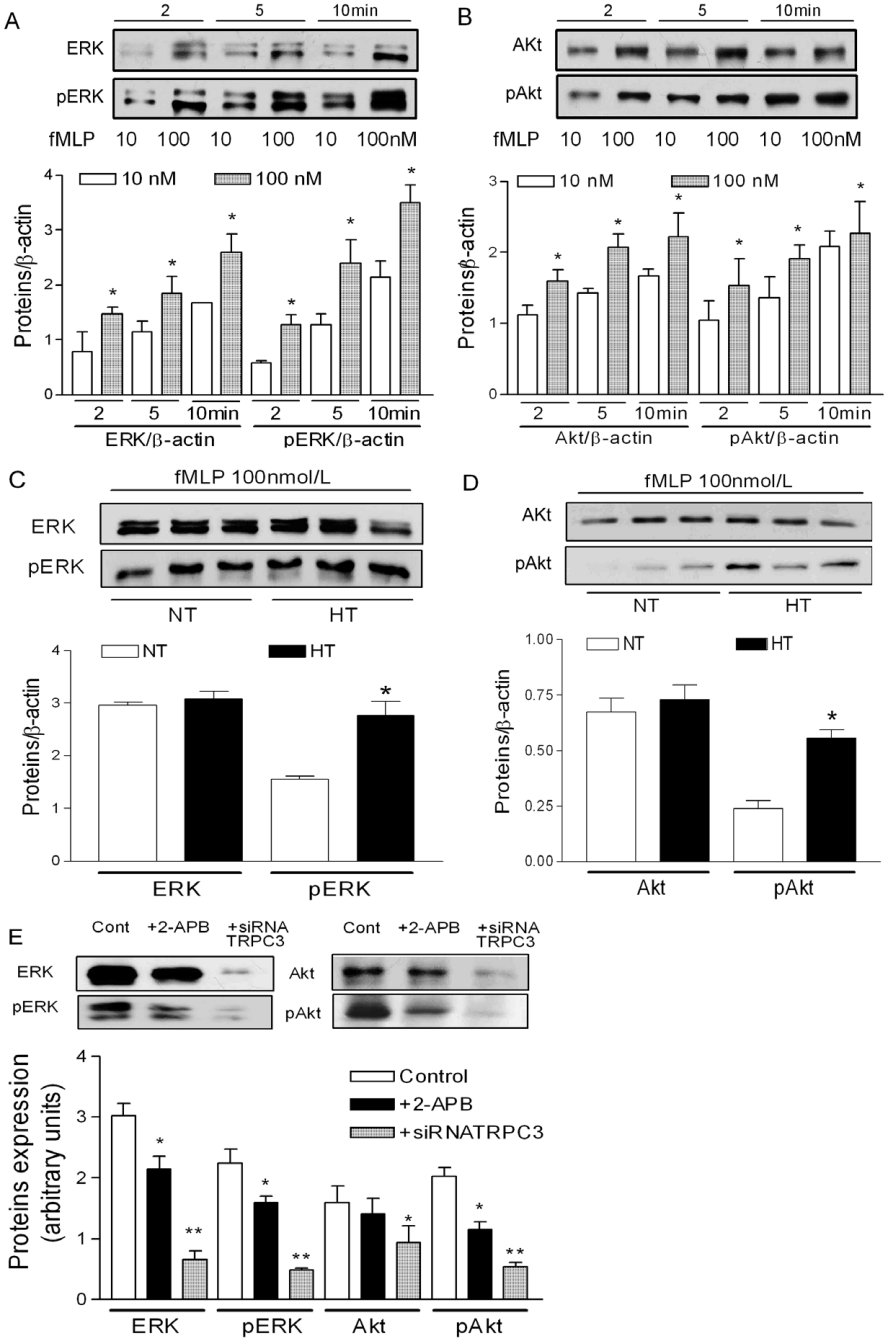
The role of Akt and ERK-dependent pathways in essential hypertension. **A**, **B**; fMLP activates ERK or phosphorylation of ERK (**A**) and Akt or phosphorylation of Akt (**B**) in a dose- and time-dependent manner in monocytes from normotensive control subjects. 10 nmol/L open bars, 100 nmol/L filled bars. Data are mean ± SEM, n = 3. *p<0.05 compared to lower concentration conditions. **C**, **D**; Increased fMLP-induced phosphorylation of ERK (**C**) and Akt (**D**) in monocytes from patients with essential hypertension. The proteins were measured using immunoblotting with specific antibodies. Data are mean ± SEM from three independent experiments. *p<0.05 compared to normotensive control subjects. **E**; fMLP activates monocytes by an ERK-dependent and Akt-dependent pathway. Akt, ERK, or pERK and pAkt were measured using immunoblotting with specific antibodies. In the presence of 2-APB or after administration of specific siRNA against TRPC3, the fMLP-induced ERK, pERK; Akt and pAkt were significantly reduced when compared with control conditions. Data are mean ± SEM from six independent experiments. *p<0.05; **p<0.01 compared to control.

We observed that fMLP activates monocytes by an ERK- and Akt-dependent pathway. As shown in **Figure 6**, administration of fMLP significantly increased phosphorylated ERK and phosphorylated Akt in a dose-dependent and time-dependent manner (**Figure 6A and 6B**). Furthermore, we compared the dose response effects of fMLP on monocytes from patients with essential hypertension and normotensive control subjects. We observed an increased phosphorylated ERK (**Figure 6C**) and phosphorylated Akt (**Figure 6D**) after fMLP stimulation of monocytes from patients with essential hypertension compared to normotensive control subjects. The expression of pERK was 2.77±0.26 vs. 1.55±0.06, n = 3, P<0.05; and the expression of pAkt was 0.56±0.04 vs. 0.24±0.04, n = 3, P<0.05 for these groups, respectively. These findings confirmed that the fMLP-induced activation of monocytes in patients with essential hypertension was associated with ERK and Akt pathways.

We observed that both the inhibition of TRPC channels using 2-APB and down-regulation of TRPC3 by specific siRNA significantly reduced the fMLP-induced expression of pERK and pAkt (**Figure 6E**). These findings underscore that the fMLP-induced activation of monocytes is TRPC3-dependent.

## Discussion

The present study showed that the increased migration of monocytes from hypertensive patients compared to normotensive control subjects could be attributed to increased expression of TRPC3 channels. After TRPC3 gene knockdown the fMLP-induced migration was similar in monocytes from hypertensive patients and normotensive control subjects.

### Patients with essential hypertension show increased activation of monocytes

The increased activation of monocytes from patients with essential hypertension has been described by several groups. Doerffel et al. reported that the secretion of IL-1beta and TNF-alpha was significantly increased in peripheral blood monocytes from hypertensive patients compared to normotensive control subjects [8]. Marketou et al. showed increased expression of angiopoietin-1 and 2 genes in peripheral monocytes with increased pulse wave velocity in patients with essential hypertension [15]. An increased monocyte chemotaxis has been observed by producing monocyte chemoattractant protein-1 through activation of nuclear factor-kappa B. An increased monocyte chemoattractant protein-1 expression could be observed in monocytes after stimulation of nuclear factor-kappa B [16]. Monocyte chemoattractant protein-1/cysteine-cysteine chemokine receptor 2 pathway appears to be involved in the increased inflammatory response observed in hypertension [9].

### Increased TRPC3 protein expression is associated with increased migration of monocytes from patients with hypertension

Chronic monocyte-mediated inflammation in arterial walls is commonly observed in hypertensive patients [17]. The migration of monocytes is an early critical step in the atherosclerotic process [18]. As indicated by several groups, an increased calcium influx causes increased migration of monocytes [19]. TRPC3 channels are cation channels mediating transplasmamembrane calcium influx [10]. Earlier studies from our group and other groups indicated that increased TRPC3 protein expression is a common finding both in patients with essential hypertension and in animal models of hypertension [1]–[5]. The present study extended these observations, showing that the increased TRPC3 protein expression may play an important role for increased activation of monocytes in patients with essential hypertension. We observed that the increased fMLP-induced migration of monocytes from hypertensive patients compared to normotensive control subjects could be attributed to increased expression of TRPC3 channels. We used fMLP to increase cytosolic calcium concentrations in monocytes confirming earlier results using that substance [20], [21]. We found that manganese influx was increased to almost 2 fold in hypertensive cells. This is in agreement with previous studies showing increased cation influx into hypertensive cells [4], [5]. Furthermore, after stimulation with fMLP the decrease of the fura-2 fluorescence by manganese quenching was more pronounced in patients with essential hypertension compared to normotensive control subjects. Both basal and agonist-activated Mn^2+^ influx were elevated in patients with essential hypertension. More importantly the fMLP-induced Mn^2+^ quenching was in agreement with an augmented fMLP-induced Ca^2+^ response in patients with essential hypertension compared to normotensive control subjects. Previous studies showed that the formyl-peptide receptor (FPR) plays a central role in the fluid shear stress response of circulating leukocytes as a mechanosensor [22]. The difference of fMLP receptor expression is almost completely absent in in mature DCs [23]. In addition, a reduced density of the extracellular domain of the FPR was reported in neutrophils from SHR [24]. Our data indicated that FPR may not be responsible for the observed differences of fMLP-induced monocytes migration between patients with essential hypertension and normotensive control subjects.

Several evidences support the notion that increased TRPC3 is associated with increased migration of monocytes from patients with essential hypertension. First, siRNA knockdown of TRPC3 significantly blocked the fMLP-induced monocyte migration. Second, the administration of the TRPC blocker 2-APB significantly reduced the migration of monocytes. Experimental data from several groups indicated 2-APB blocks TRPC channels [17], [18], [19]. We measured spontaneous migration as medium without agonist (Control), out data indicating that spontaneous migration is not affected by 2-APB. Spontaneous migration and agonist-induced migration may be affected by several pathways. For example, previous reports showed that inhibitors including forskolin plus 3-isobutyl-1-methylxanthine reduced spontaneous migration as well as agonist-induced migration [25]. Liu et al. showed that 2-APB abolished spontaneous Ca^2+^ transients [26]. 2-APB partially inhibited LPC-mediated activation of non-selective cation currents and chemotaxis in monocytes, indicating that activation of non-selective cation channels may be required for migration of LPC-stimulated monocytes [27]. 2-APB diminished the serum-induced increase of Ca^2+^ waves and inhibited cell proliferation [28]. Specificity of TRPC3 blockers has been questioned in the past. Therefore we also investigated the migration of monocytes after downregulation of TRPC3 using specific siRNA. We observed an increased fMLP-induced migration of monocytes from hypertensive patients compared with normotensive control subjects. After siRNA knockdown of TRPC3 the fMLP-induced migration was similar in monocytes from patients with essential hypertension and normotensive control subjects. Our experimental data using the inhibitor 2-APB were in line with our findings using siRNA for TRPC3 knockdown. Both, experiments using the inhibitor 2-APB, as well as specific TRPC3 knockdown using the siRNA technique supported the notion that increased monocyte migration in patients with essential hypertension is associated with increased TRPC3 channels. Our study confirms that TRPC3 does not affect spontaneous migration but only agonist-induced migration in monocytes. It may indicate that particularly the agonist-induced migration is augmented by increased TRP channels in the patients with essential hypertension.

### Which mechanisms mediate increased migration of monocytes from patients with hypertension?

An important step for induction of migration is the phosphorylation of proteins involved in cell locomotion [29]. The present study indicates that fMLP significantly increased phosphorylated Akt and phosphorylated ERK in a dose- and time-dependent manner. Both phosphorylated Akt and phosphorylated ERK have been associated with increased cell migration. The activation of Akt and ERK subsequently enhances phosphorylation of the regulatory myosin light chain and finally migration [30], [31]. Our data provide the first direct evidence for the involvement of PI3K, ERK and Akt signaling pathways of chemotaxis for monocytes migration in the patients with essential hypertension. The specificities of genistein or wortmannin have been questioned. Genistein may inhibit protein tyrosine kinases, bind to PPARγ, and affect potassium channels. Wortmannin may inhibit PI3K, inhibit polo-like kinase and affect myosin light chain kinase. Therefore our results using genistein and wortmannin can not exclude that signal transduction pathways other then tyrosine kinases and PI3K may be involved [32]–[35]. As suggested by Noma et al. the activation of both ERK and PI3K/Akt pathways constitute major signaling events for activation of monocytes, but not lymphocytes [36].

Our study indicated that the fMLP-induced migration was significantly different between hypertensive patients and normotensive subjects, and the fMLP-induced phosphorylated ERK and phosphorylated Akt responses were elevated in hypertensive patients. These findings may indicate that ERK- and Akt-related pathways mediated the effects on elevated migration in hypertension. Our study showed increased migration in monocytes from hypertensive patients associated with increased TRPC3 protein expression. Increased migration of monocytes may aggravate atherosclerosis in hypertensive patients. It may be speculated that reduction of blood pressure as well as amelioration of increased TRPC3 expression in monocytes may be important goals in order to prevent hypertensive complications.

## Methods

### Patient characteristics

The clinical and biochemical characteristics of normotensive control subjects and patients with essential hypertension are presented in **Table 1**. Control subjects and patients with essential hypertension were on unrestricted normal diet with no sodium restriction. A diagnosis of hypertension was based on systolic blood pressure 140 mmHg or a diastolic (Korotkoff phase V) blood pressure 90 mmHg obtained by conventional sphygmomanometric methods. Blood pressure was obtained on three occasions in a sitting position after a rest of 10 minutes. No patient had significant pain, elevated temperature, other acute conditions, or antihypertensive medication. Furthermore, C-reactive protein was normal in all subjects. Diagnosis of essential hypertension was established after exclusion of secondary forms of hypertension. Normotensive control subjects were recruited among patients with minor complaints. Subjects with major medical illness were excluded. All subjects gave written informed consent and the study was approved by the local ethics committee.

**Table 1:** Clinical and biochemical characteristics of normotensive control subjects and patients with essential hypertension.

Characteristic	Normotensive control subjects	Patients with essential hypertensive
Ages (years)	63±5	65±7
N (men/women)	12/13	14/12
SBP (mmHg)	125±7	159±8*
DBP (mmHg)	77±10	93±13*
Body mass index (kg/m^2^)	24±5	28±4*
Waist circumference (cm)	79±6	88±6*
Heart rate (per minute)	73±9	78±10
Hemoglobin (g/dl)	138±11	135±16
Serum sodium (mmol/l)	139±5	140±6
Serum potassium (mmol/l)	3.70±0.50	4.00±0.50
Cholesterol (mmol/l)	4.70±0.80	5.21±1.09
Triglycerides (mmol/l)	0.98±0.36	1.39±0.68
LDL (mmol/l)	2.80±0.25	2.73±0.64
HDL (mmol/l)	1.62±0.34	1.56±0.36
FPG (mmol/l)	5.59±0.64	5.97±0.62
Serum creatinine (µmol/l)	74±20	82±7
h-sensitivity CRP (mg/L)	1.28±0.69	1.51±0.44

SBP: Systolic blood pressure; DBP: Diastolic blood pressure; LDL: Low-density cholesterol; HDL: High-density cholesterol; FPG: Fasting plasma glucose; Data are mean ± SD.

* P<0.01, compared with normotensive control subjects.

### Preparation of cells

Human monocytes were obtained from heparinized blood using superparamagnetic polystyrene beads coated with a primary monoclonal antibody specific for the CD14 membrane antigen expressed on human monocytes [37]. (Dynal Biotech, Hamburg, Germany) and resuspended in Hanks balanced salt solution containing: NaCl, 136 mmol/l; KCl, 5.40 mmol/l; KH_2_PO_4_, 0.44 mmol/l; Na_2_HPO_4_, 0.34 mmol/l; D-glucose, 5.6 mmol/l; CaCl_2_, 1 mmol/l; N-2-hydroxyethyl-piperazine-N0-2-ethanesulfonic acid (HEPES), 10 mmol/l; pH 7.4.

### Fluorescence measurements of cations

For ratiometric imaging experiments, cells were loaded with 2 µmol/l calcium-sensitive, cell-permeable, intracellular fluorescence dye 1-[2-(5-carboxyoxazol-2-yl)-6-aminobenzofuran-5-oxy]-2-(20-amino-50-methylphenoxy)-ethane-N,N,N0,N0-tetraacetic acid pentaacetoxy methyl ester (fura2/AM; Merck Biosciences, Darmstadt, Germany) at room temperature for 60 min and washed to remove extraneous dye as previously described [4]. Fluorescence measurements were performed at 510 nm emission with excitation wavelengths of 340 nm and 380 nm (Fluoroskan Ascent Fluorometer; Thermo Lab-Systems Oy, Helsinki, Finland). Quenching of fura-2 fluorescence with manganese (1 µmol/L), Fura-2 fluorescence in monocytes was monitored at 510 nm using the isobestic excitation wavelength of 360 nm. Each measurement was performed for 300 s; manganese was added after 20 s and a stimulus (fMLP 100 nmol/L) after 150 s. For each curve obtained in the presence of a stimulus, the difference to a curve obtained in the absence of a stimulus was calculated, and yielding the corrected quenching curve [12], [13]. Control experiments showed no significant dye leakage out of the cells.

### Quantitative determination of TRPC protein expression using in-cell western assay

Quantitative in-cell Western assays of TRPC3 channels in monocytes were performed using the Odyssey infrared imaging system (Licor Biosciences, Bad Homburg, Germany) according to the manufacturer's recommendations [38]. Removed growth media manually and monocytes immediately fixed with fixing Solution (3.7% formaldehyde in 1× PBS) for 20 minutes at room temperature (RT). For permeabilization, cells were washed five times with 1× PBS containing 0.1% Triton X-100 for 5 minutes. After that incubated with rabbit anti-TRPC3 antibodies (1∶1000, Alomone Laboratories, Jerusalem, Israel) as the primary antibodies for 2 h, washed, incubated with IRDye 800 CW infrared fluorescent dye conjugated goat anti-rabbit antibodies (1∶1000, Biomol, Hamburg, Germany) as the secondary antibody overnight and washed, and quantitative imaging was performed at 810 nm emission with an excitation wavelength of 780 nm. Control experiments were performed by the omission of cell permeabilization and omission of the primary or secondary antibodies. For internal reference we used mouse anti-CD14 antibodies and Alexa Fluor680-allophycocyanin fluorescence–labeled goat anti-mouse antibodies (1∶1000, MoBiTec, Göttingen, Germany). Imaging was performed at 700 nm emission with an excitation wavelength of 680 nm. Additional measurements were performed in the absence of cell permeabilization to investigate TRPC channel protein expression on the cell surface.

### Small interfering RNA knockdown of TRPC3

RNA interference for the down-regulation of a specific gene in living cells by small interfering RNA (siRNA) was performed. Human monocytes were transfected with siRNA specific for TRPC3 for 24 or 48 h using a silencer siRNA transfection kit (Ambion, Austin, Texas, USA) or with negative control siRNA. Briefly, monocytes were resuspended in Advanced DMEM (Dulbecco's Modified Eagle Medium, Invitrogen) with 10% foetal bovine serum and incubated with siPORT amine (Ambion) and 1 µl chemically synthesized siRNA (final concentration, 20 nmol/l; Ambion) specific for the respective TRPC channel. The target sequence for TRPC3 was 5′ –GGUUAAACCUCUUCACUCAtt–3′ (sense) and 5′–UGAGUGAAGAGGUUUAACCtg–3′ (antisense).

Monocytes were then investigated using in-cell western assays and fluorescence measurements as described above. In control experiments using the described transfection procedure, negative control siRNA (Ambion) that has no significant homology to any known human gene sequence did not affect GAPDH expression (100±2%; n = 4).

### Immunoblotting of TRPC3, Akt, pAkt, ERK and pERK proteins expression

Immunoblotting was performed as previously described by our group [1]. Monocytes were isolated and resuspended in ice-cold lysis buffer. Cells were lysed in high-salt extraction buffer (0.5 mol/L Tris, 1% NP-40, 1% Triton X-100, 1 g/L sodium dodecyl sulfate, 1.5 mol/L NaCl, 0.2 mol/L EDTA, 0.01 mol/L EGTA) and 0.2 mmol/L protease inhibitor, placed at −20°C for 20 min and centrifuged at 12,000 g at 4°C for 20 min to remove insoluble material. Protein concentration was determined using a DC protein assay kit (Bio-Rad, Hercules, CA, USA). Fifty-µg portions of the protein were resolved on SDS–polyacrylamide gels (10%) and electroblotted onto polyvinylidene difluoride membranes. After transfer, the membranes were blocked for 4 hours at room temperature in blocking buffer (Bio-Rad). The membranes were incubated with primary antibodies against TRPC (alomone labs; 1∶200), Akt, pAkt, ERK, and pERK (Santa Cruz Biotechnology; 1∶500) followed by incubation with secondary horseradish peroxidase-conjugated secondary antibody. The specific bands were quantified using an image analyzer (bio-rad laboratories, Hercules, CA, USA). The specificity of TRPC3 antibodies was tested using antigen competition experiments [37].

### Monocyte migration assay

The monocytes chemotaxis assays were performed using a modified 48-well Boyden microchemotaxis chamber (Neuroprobe, Inc; Gaithersburg, MD; USA) according to the manufacturer's recommendations and reports from the literature [39], [40]. Wells covered with 8 µm-pore size polycarbonate membranes (Neuroprobe, Inc) separated the upper and the lower chambers. The camber was assembled, monocytes were resuspended in RPMI 1640 containing 0.5% BSA. 50 µl of the cell suspension (1×10^6^ cells/mL) was placed into the upper compartment of the chemotaxis chamber, and monocytes were allowed to migrate toward chemoattractant formylated peptide Met-Leu-Phe (fMLP, 100 nmol/L; Sigma-Aldrich) or tumor necrosis factor-α (TNF-α, 100 ng/mL, Sigma-Aldrich) which was placed in the lower chamber for 4 or 48 hours at 37°C in a humidified atmosphere (5% CO_2_). After the incubation time the polycarbonate filter membranes were dehydrated, stained using fura2-AM and fluorescence was detected at 510 nm emission with 360 nm excitation, which is the calcium-independent fura-2 excitation wavelength. Monocytes chemotaxis was quantified by calculating the ratio of marginal and centrally located fluorescence. Negative controls were performed where RPMI 1640 medium was placed in the lower chamber. The experiments using a rapid endotoxin test (Pyrosate, sensitivity 0.25 EU/ml, East Falmouth, Massachusetts, USA) showed that fura2-AM was free of endotoxin. After 1-hour incubation, gently inversion of the tubes was performed until the occurrence of a solid gel-clot. The PPC (Positive Product Control) tubes showed the formation of a hard gel (clot+), but SPL (Sample) tubes did not clot. We made serial dilutions for Fura2-AM solutions (final concentrations, 1 µmol/l, 2 µmol/l, and 4 µmol/l). SPL tubes did not show any clot+ (i.e., the sample contains less than 0.25 EU/ml of endotoxin).

In addition, after the incubation time the polycarbonate filter membranes were fixed, dehydrated, stained using Harris haematoxylin, and mounted on a glass slide. Chemotaxis of monocytes was quantified by counting the number of cells that had completely migrated through the membrane into six random squares per well (magnification ×40). Chemotaxis of monocytes was expressed as chemotaxis ratio, which is the ratio between the cells migrated towards the chemoattractant and those migrated towards control medium into the polycarbonate filter membranes. Negative controls were performed where RPMI 1640 medium was placed in the lower chamber. All experiments were performed at least in triplicate. All data on fMLP-induced migration and TNF-α-induced migration were analyzed based on the difference (% of own medium control) in normotensive control subjects and patients with hypertension.

### Monocytes of pseudopod extension

To evaluate the effects of fMLP-induced monocytes shape changes, monocytes were seeded in the cover glass and held in the round steel chamber filled with 50 µl PBS. fMLP (final concentration 100 nM) was added onto cover glass, which initiated the extension of the cells pseudopod. Pseudopod extension was observed by means of an inverted Nikon (TE2000U, Tokyo, Japan) microscope equipped with a 40× objective. The microscope images were recorded with a charge-coupled device (CCD) camera (Nikon, DS-5MC). A real-time counter and recorded images were analyzed with Nikon-Elements Imaging Software (NIS-BR3.2), and the rate of pseudopod extension was calculated from the measured pseudopod lengths and time lapses. The nonresponding fraction of monocyts was not reported in the averages of the rates of pseudopod extension.

### Flow Cytometric Determination of monocyte subset

100 µl of heparinized blood samples were stained with anti-human CD14 conjugated with phycoerythrin (CD14-PE), anti-human CD16 conjugated with fluorescein isothiocyanate (CD16-FITC) antobodies (BD Biosciences, San Diego, Calif., USA) for 15 min at room temperature, and following lysis and washing, flow-cytometric detection of monocyte subsets was performed (FACS Caliber; BD Biosciences). Fifty thousand cells were analyzed from each sample and the percentage and number of cells out of the total monocytes were compared.

### Statistics

All data were expressed as mean ± SEM and were compared using a two-tailed Student's t-test. The null hypothesis was rejected at p<0.05. Where error bars do not appear on the figure, error was within the symbol size.
